# OpenSeismoMatlab: A new open-source software for strong ground motion data processing

**DOI:** 10.1016/j.heliyon.2018.e00784

**Published:** 2018-09-26

**Authors:** George Papazafeiropoulos, Vagelis Plevris

**Affiliations:** a Institute of Structural Analysis and Antiseismic Research, National Technical University of Athens, Heroon Polytechniou 9, Zografou Campus, 15780, Athens, Greece; b Department of Civil Engineering and Energy Technology, OsloMet—Oslo Metropolitan University, Pilestredet 35, 0166, Oslo, Norway

**Keywords:** Civil engineering, Computer science, Natural hazards, Structural engineering

## Abstract

OpenSeismoMatlab is an innovative open-source software for strong ground motion data processing, written in MATLAB. The software implements an elastoplastic bilinear kinematic hardening constitutive model and uses a state-of-the-art single step single solve time integration algorithm featuring exceptional speed, robustness and accuracy. OpenSeismoMatlab can calculate various time histories and corresponding peak values, Arias intensity and its time history, significant duration, various linear elastic response spectra and constant ductility inelastic response spectra, as well as Fourier amplitude spectrum and mean period. Due to its open-source nature, the software can be easily extended or modified, having high research and educational value for the professional engineering and research community. In the present paper, the structure, algorithms and main routines of the program are explained in detail and the results for various types of spectra of 11 earthquake strong ground motions are calculated and compared to corresponding results from other proprietary software.

## 1 Introduction

Earthquake resistant building codes require earthquake engineering studies which, in order to be performed, need strong motion records as original input data. It is therefore important to make realistic selections and processing of the raw input strong motion records in order to calculate the seismic parameters which will help in the estimation of the dynamic response of the structure(s) to be designed. Various software programs have been developed for the selection of the strong ground motions which are used for the dynamic analysis and design of structure(s) [1, 2]. Among the most important seismic parameters of a strong ground motion are the various types of spectra (i.e. elastic response spectrum, constant ductility spectrum, constant-damage yield strength spectrum, Fourier spectrum, etc.) which result from the processing of the ground motion and which are used in various seismic design procedures, such as the Dynamic Response Spectrum Analysis (DRSA), the Uncoupled Modal Response History Analysis (UMRHA), the Modal Pushover Analysis (MPA) procedures for dynamic analysis [3]. Furthermore, by adjusting the Fourier spectrum of a strong ground motion, it is possible to control its frequency content. Therefore, the use of a robust and accurate strong motion processing software is critical for the proper seismic design of structures, including strategies for designing earthquake-resistant buildings to ensure the health, safety, and security of building occupants and assets during the structure's lifetime.

The concept of the elastic response spectrum was introduced by G.W. Housner [4], whereas [5] is a fundamental work on linear elastic response spectra. Since then a large research effort has been made for the evaluation of the seismic response of linear SDOF systems with particular attention to the effect of input motion and site conditions. Most studies on inelastic response spectra have focused on the selection of the elastic-perfectly plastic material behavior, on taking into account the effects of the duration of the motion and on scaling methods [6, 7, 8, 9]. In addition, [10] was among the first studies that systematically investigated the elastic and inelastic structural response to pulse-like excitations (typically not caused by earthquakes).

Many software programs, either free or commercial, have been developed for the processing of strong ground motion data. Some characteristic cases are presented below:

• **SMA** (Strong Motion Analyst Processing Software) is a commercial Windows-based tool designed to interactively process strong motion accelerograms, featuring instrument correction, data editing, filtering, ground motion integrations, Fourier and Response Spectra calculations, and V1, V2, V3 file format output. It has been developed by the Kinemetrics company.
• **EQ-TOOLS** (latest version is 3.0) is a free closed source software for earthquake engineering education which allows the user to select, analyze, scale, and modify ground motions. The capabilities of selection and analysis as well as scaling of ground motion records against several types of target spectra, including the ASCE 7 spectrum, spectra from attenuation relationships, and conditional mean spectra are included. Ground motion history analysis, linear response spectrum analysis and Fourier amplitude analysis and a module that enables the modification of ground motions for consideration of site effects are provided. It has been developed by the George E. Brown, Jr. Network for Earthquake Engineering Simulation (NEES).
• **PRISM** (Processing and Review Interface for Strong Motion Data) is a free open-source software used for processing strong-motion records [11, 12, 13, 14]. It can be installed and run as a stand-alone system on common operating systems such as Linux, Mac and Windows and is flexible and extensible to incorporate new strong motion processing techniques. It includes capabilities for modification, correction, scaling, truncation and baseline correction of earthquake records and it can calculate a variety of strong motion parameters (Arias intensity, elastic and inelastic response spectra, acceleration, velocity, displacement and force-displacement response histories). Various hysteresis models are provided (linear elastic, bi-linear, tri-linear, modified Takeda, Bouc-Wen, and Al-Bermani)
• **SEISMOSIGNAL** is an interesting, user-friendly and efficient commercial software for processing of strong motion data [15]. Among others, it can calculate the elastic, constant ductility, Fourier amplitude and power spectra and it provides for filtering of high and low frequency record content and estimation of other important seismological parameters, such as the Arias Intensity and the significant and effective durations.
• **OPENSIGNAL** is a free closed-source software platform for the processing and selection of seismic records, signal processing, response spectra analysis, soil spectra analysis and more [16, 17]. It provides filtering uncorrected ground motion records and calculation of the main parameters of a record (Arias Intensity, duration, PGA, PGV, elastic response spectra, etc.).
• **USDP** (Utility Software for Data Processing) is a computer program that can be used for strong ground-motion data processing by various filtering and baseline adjustment techniques and spectral calculations (Linear spectral analysis, Fourier spectral analysis, Constant strength, ductility and base-shear coefficient nonlinear spectral analysis) for a variety of stiffness and/or strength degrading hysteretic models [18]. It has been developed by the METU-Earthquake Engineering Research Center team and uses public-open Fortran source codes.
• **TSPP** (Time Series Processing Programs) is a collection of FORTRAN programs that have been developed for processing and manipulating strong-motion accelerograms in terms of displacement, velocity and acceleration time-histories, response and Fourier spectra and filtering [19].
• **VIEWWAVE** (v2.2.0) is a free closed source software for processing and viewing strong motion records. It can read a large variety of files in many formats and can calculate various waveforms, Fourier and power spectrum, as well as acceleration, velocity and displacement response spectra [20].

Apart from the above software, some other rather elementary programs have been developed in MATLAB programming language [21, 22, 23, 24, 25]. However, none of these MATLAB implementations contains advanced time integration algorithms for the extraction of the displacement, velocity and acceleration time histories and the various response spectra. In the present study, a new MATLAB open-source software, called **OpenSeismoMatlab**, is presented which, compared to other similar software, has the following advantages and unique characteristics:

• It uses state-of-the-art time integration algorithms which are more robust and accurate [26, 27] compared to conventional integration techniques (Newmark, etc.) that are widely used by other software for strong motion data processing. The former algorithms belong to a general single step single solve family and can be adjusted through the specification of 14 independent integration constants to control numerical dissipation and dispersion, continuity of acceleration, and the order of overshooting in displacement and velocity. By adjusting a number of parameters, the user can select from a large family of time integration algorithms, according to [27] and this permits the manual configuration and optimization of the quality of the desired results (time histories, spectra, etc.).
• It is completely free and provided together with its source code (open source). OpenSeismoMatlab is of high educational value, since it contains simply written MATLAB code with comments and is generally easy to be understood by the user. The rationale of the implemented methods is explained in detailed comments within the code. Apart from this, the open-source code format provides the opportunity of extending/upgrading or integrating the software in all possible ways.
• Furthermore, the elastoplastic bilinear kinematic hardening constitutive model which is fundamental for the computation of the nonlinear spectra is accurately formulated and programmed in the software. No simplified versions of the elastoplastic bilinear kinematic hardening constitutive model [28, 29, 30] are used, as is usually the case in the literature.

In the following sections, OpenSeismoMatlab is presented, and then it is applied in a number of earthquake records for the calculation of the various response spectra and other quantities.

## 2 Materials & methods

In this section the design of the various algorithms used by OpenSeismoMatlab for the computation of the strong motion data processing results (spectra, time histories, etc.) are given. For each algorithm the structure of the main MATLAB function and its subroutines (if present) are provided and the architecture of the various MATLAB codes within the OpenSeismoMatlab software is presented. Finally, the capabilities and restrictions of OpenSeismoMatlab are discussed. OpenSeismoMatlab can calculate the following strong motion data processing output:

• Time history of velocity
• Time history of displacement
• Peak ground acceleration (PGA)
• Peak ground velocity (PGV)
• Peak ground displacement (PGD)
• Time history of normalized Arias intensity ($\bar{AI}$)
• Total Arias intensity
• Time interval between 5% and 95% of Arias intensity has occurred (significant duration D_5_95_)
• Linear elastic pseudo-acceleration (PSA) response spectrum
• Linear elastic pseudo-velocity (PSV) response spectrum
• Linear elastic displacement (SD) response spectrum
• Linear elastic velocity (SV) response spectrum
• Linear elastic acceleration (SA) response spectrum
• Constant ductility inelastic displacement response spectrum
• Constant ductility inelastic velocity response spectrum
• Constant ductility inelastic acceleration response spectrum
• Fourier amplitude spectrum
• Mean period (T_m_)

The source code of OpenSeismoMatlab has been uploaded on two different distribution channels: (i) the File Exchange service of MATLAB central [31]; and (ii) on ResearchGate [32], so that it is publicly available. The source code is organized in folders as follows:

• A folder named “data” contains the acceleration time histories of the earthquakes considered in this study, in digitized format
• A folder named “examples” contains three MATLAB scripts which illustrate how the software can be used properly for the generation of Elastic Response Spectra, Fourier Spectra and Constant Ductility Response Spectra
• A folder named “figures” contains the figures that are generated after the execution of the MATLAB scripts contained in the “examples” folder
• A folder named “lib” contains all the subroutine functions of the OpenSeismoMatlab software. These functions are substantial for the application of OpenSeismoMatlab.

**Fig. 1:**
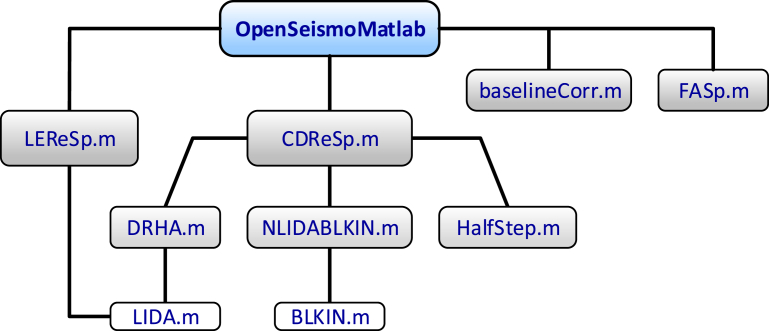
Schematic dependency diagram between the various functions included in the OpenSeismoMatlab package.

In Fig. 1 the dependency diagram between the various functions included in the OpenSeismoMatlab package is shown. The four main functions are LEReSp for the linear elastic response spectra, CDReSp for the constant ductility response spectra, FASp for the Fourier spectra and baselineCorr for the baseline correction of the input ground motion. The functions DRHA, NLIDABLKIN and HalfStep are called directly by the function CDReSp and are used for Dynamic Response History Analysis, NonLInear Dynamic Analysis with BiLinear KINematic hardening model, and reproduction of an earthquake excitation with the half time step, respectively. The function LIDA is used for Linear Incremental Dynamic Analysis and is called by the functions LEReSp and DRHA, whereas the function BLKIN is called by the function NLIDABLKIN.

The beginning section of the main MATLAB function (OpenSeismo.m) is shown in the following code segment for purposes of completeness, and to show how the various variables that appear in parts of the main function code presented in the subsequent sections are defined. The two necessary input arguments are the time column vector (denoted as time) and the ground acceleration time history column vector (denoted as xgtt). Apart from these, the various default values which are set in the required variable definitions in case they are not specified by the user are shown. These are the ratio of critical viscous damping *ξ* (denoted in the code as ksi), the lower period limit *T*_*1*_, the upper period limit *T*_*2*_ and the period step *dT* of the generated response spectra (denoted in the code as T1, T2 and dT respectively), and finally the target ductility ratio *μ*_*t*_ (denoted in the code as mu).

**Table:** 

function seismic=OpenSeismo(time,xgtt,varargin)
%% Initial checks
if nargin<2
error(‘Input arguments less than required’)
end
if nargin>7
error(‘Input arguments more than required’)
end
% set defaults for optional inputs
optargs = {0.05,0.04,10,0.05,2};
% skip any new inputs if they are empty
newVals = cellfun(@(x) ∼isempty(x), varargin);
% overwrite the default values by those specified in varargin
optargs(newVals) = varargin(newVals);
% place optional args in memorable variable names
[ksi,T1,T2,dT,mu] = optargs{:};
time = time(:);
xgtt = xgtt(:);
dt = time(2)-time(1);

**1** **Displacement and velocity time histories and peak values**

This part of the OpenSeismoMatlab code is quite straightforward. The MATLAB code shown below is executed in order to determine the displacement time history of the input motion, the velocity time history, the peak displacement, the peak velocity and the peak acceleration. The time integration is simply performed by the summation of the product of the integrand function by the time step *Δt*. The velocity and displacement time histories are given by Eqs. (1) and (2) respectively:

(1) $${\dot{u}}_{g}^{k}=\left(\sum_{i=1}^{k}{\ddot{u}}_{g}^{i}\right)\Delta t$$

(2) $$u_{g}^{k}=\left(\sum_{i=1}^{k}{\dot{u}}_{g}^{i}\right)\Delta t$$

where k denotes the k^th^ time step of the earthquake motion. Since the size of the time step is constant throughout the various earthquake motions, Eqs. (1) and (2) make use of the cumulative sums (cumsum) of the earthquake acceleration and the earthquake velocity respectively. The peak values are given by the Eqs. (3), (4), and (5) respectively:

(3) $$PGA=\max\left(\left|{\ddot{u}}_{g}\right|\right)$$

(4) $$PGV=\max\left(\left|{\dot{u}}_{g}\right|\right)$$

(5) $$PGD=\max\left(\left|u_{g}\right|\right)$$

The user has the option to perform baseline correction to the input acceleration data, if desired, as shown in the following code:

**Table:** 

% TIME SERIES
if baselineSw
[cor_xg,cor_xgt,cor_xgtt] = baselineCorr(time,xgtt);
seismic.acc=cor_xgtt;
seismic.vel=cor_xgt;
seismic.disp=cor_xg;
else
% Acceleration time history
seismic.acc = xgtt;
% Velocity time history
seismic.vel = cumtrapz(time,xgtt)*dt;
% Displacement time history
seismic.disp = cumtrapz(time,seismic.vel)*dt;
end

% PEAK RESPONSES
% Peak ground acceleration
seismic.PGA = max(abs(xgtt));
% Peak ground velocity
seismic.PGV = max(abs(seismic.vel));
% Peak ground displacement
seismic.PGD = max(abs(seismic.disp));

This capability is activated if the boolean variable baselineSw is set equal to true. If so, the function baselineCorr.m is used for this purpose. The source code of baselineCorr.m is shown in the following code:

**Table:** 

function [cor_xg, cor_xgt, cor_xgtt] = baselineCorr(time,xgtt)
dt = time(2)-time(1);
% Least squares fit through acceleration history
p=polyfit(time,xgtt,1);
lsf_cor_xgtt = polyval(p,time);
cor_xgtt1 = xgtt - lsf_cor_xgtt;
% Integrate for velocity
un_xgt = cumtrapz(time,cor_xgtt1)*dt;
% Least squares fit through velocity history
ca2 = polyfit(time,un_xgt,1);
cor_xgtt = cor_xgtt1 - ca2(1);
% Corrected velocity
cor_xgt = cumtrapz(time,cor_xgtt)*dt;
% Corrected displacement
cor_xg = cumtrapz(time,cor_xgt)*dt;

The function baselineCorr.m performs linear baseline correction for an uncorrected acceleration time history. Initially, first order fitting (straight line) is performed and the fitting line is subtracted from the acceleration time history, giving thus the first correction. Afterwards, this first correction of the acceleration is integrated to obtain the velocity, and then first order fitting (straight line) is reapplied on this velocity time history. The gradient of the straight fitting line is then subtracted from the first correction of the acceleration time history, giving thus the second correction of the acceleration time history. The second correction of the acceleration time history is then simply and doubly integrated to give the corrected velocity and displacement time histories, respectively.

**2** **Arias Intensity**

The Arias Intensity was proposed as an intensity measure of an earthquake by [33, 34], since it was recognized that the peak values alone cannot adequately portray the intensity of a ground motion. It is broadly defined as the sum of the energies per unit mass, dissipated due to the ground motion, by a population of Single Degree of Freedom (SDOF) systems with all natural frequencies. For undamped linear elastic SDOF systems, it can be shown [33, 34] that the Arias Intensity (AI) is given by Eq. (6):

(6) $$AI=\frac{\pi }{2}\int_{0}^{t_{f}}{\ddot{u}}_{g}^{2}\left(t\right)dt$$

where *t*_*f*_ is the total duration of the earthquake. For a digitized strong motion data, Arias intensity is given by Eq. (7):

(7) $$AI=\frac{\pi }{2}\left(\sum_{i=1}^{i_{\max}}{\left({\ddot{u}}_{g}^{i}\right)}^{2}\right)\Delta t$$

where *i*_*max*_ denotes the total number of time increments of the earthquake motion. OpenSeismoMatlab can also output the time history of the normalized Arias Intensity, which expresses how the current ${\bar{AI}}_{k}$ (up to the current time step k, normalized with the total AI) evolves with time during the earthquake motion, as given by Eq. (8):

(8) $${\bar{AI}}_{k}=\frac{AI_{k}}{AI}=\frac{\frac{\pi }{2}\left(\sum_{i=1}^{k}{\left({\ddot{u}}_{g}^{i}\right)}^{2}\right)\Delta t}{AI}$$

where *AI*_k_ is the value of the Arias Intensity at the k^th^ time step of the earthquake motion. The above calculations is performed by the following MATLAB code:

**Table:** 

% ARIAS INTENSITY
% time history of Arias Intensity
aint2 = cumsum(xgtt.ˆ2)*pi*dt/2;
% Total Arias Intensity at the end of the ground motion
arias = aint2(end);
seismic.arias = arias;
% time history of the normalized Arias Intensity
seismic.aint2 = aint2/arias;

**3** **Significant duration**

The definition of significant duration is given in [35, 36]. In this definition, the significant duration is defined as the time interval between the time at which 5% of the seismic energy is attained and the time at which 95% of the seismic energy is attained. It is denoted as D_5_95_. The computational implementation is given in the MATLAB code shown below. The code outputs both the significant duration (denoted in the code as D_5_95) and the time instants at which 5% and 95% of AI are attained (arranged in a row vector denoted in the code as t_5_95).

**Table:** 

% SIGNIFICANT DURATION
% elements of the time vector which are within the significant duration
timed = time(aint2>=0.05*arias & aint2<=0.95*arias);
% starting and ending points of the significant duration
seismic.t_5_95 = [timed(1),timed(end)];
% significant duration
seismic.D_5_95 = timed(end)-timed(1);

**4** **Elastic Response Spectrum**

The Linear Elastic Response Spectrum (LEReSp) for a response quantity (acceleration, velocity, displacement, etc.) is a plot of the peak value of the quantity as a function of the natural vibration period (T_n_) or eigenfrequency (f_n_) of a population of linear elastic SDOF systems. Each linear elastic response spectrum is associated with a fixed damping ratio ξ. A flowchart of the calculation of the linear elastic response spectrum of an earthquake strong ground motion is shown below:

**Table:** 

**Input:**${\ddot{u}}_{g}$,ω,ξ
InitializeSD, SVSA
Set$u_{0}$ and ${\dot{u}}_{0}$
for each SDOF *i* with eigenfrequency$\omega_{i}$
if$\omega_{i}\Delta t/\left(2\pi \right)>0.02$
Reproduce${\ddot{u}}_{g}$with half time step (fromΔttoΔt/2)
SetΔt=Δt/2
end
Perform dynamic analysis of SDOF with input (${\ddot{u}}_{g}$,ξ,$u_{0}$,${\dot{u}}_{0}$)
Assignmax(|u(t)|)toSD(i)
Assign$\max\left(\left|\dot{u}\left(t\right)\right|\right)$toSV(i)
Assign$\max\left(\left|\ddot{u}\left(t\right)\right|\right)$toSA(i)
end
CalculatePSV=ωSDand$PSA=\omega^{2}SD$
**Output:**SD,SV,SA,PSV,PSA

It is noted that all time integration algorithms require the use of relatively small time-steps in order to deliver sufficiently accurate solutions [37, 38]. For this purpose, a maximum value of the ratio between the integration time-step and the period of the oscillator being analyzed is imposed, as shown in the above flowchart ($\max\left\{\omega_{i}\Delta t/\left(2\pi \right)\right\}=0.02$), where *ω*_*i*_ is the circular eigenfrequency of the *i*^*th*^ oscillator. Initially, the program uses the time step of the input acceleration time history as the time step of the dynamic analysis, and then if this is found to violate the aforementioned maximum, the algorithm automatically reproduces the acceleration time history with half the current time step through linear interpolation, so that the threshold value is respected. The maximum limit that is specified above (0.02) leads to sufficiently accurate solutions. However, in OpenSeismoMatlab it can be changed manually by the user, if required, in order to handle special cases. The MATLAB function that reproduces the acceleration time history with half the time step is called HalfStep and its code is shown in the following:

**Table:** 

function uNew = HalfStep(u)
a=[([0;u(1:end-1)]+u)/2,u]';
uNew=a(:);
uNew(1)=[];
end

The main OpenSeismoMatlab function for the calculation of the linear elastic response spectrum is called “LEReSp” and its source code is shown in the following:

**Table:** 

function [PSa,PSv,Sd,Sv,Sa]=LEReSp(dt,xgtt,T,varargin)
% set defaults for optional inputs
optargs = {0.05,0.01,‘U0-V0-CA’,0};
% skip any new inputs if they are empty
newVals = cellfun(@(x) ∼isempty(x), varargin);
% overwrite the default values by those specified in varargin
optargs(newVals) = varargin(newVals);
% place optional args in memorable variable names
[ksi,dtTol,AlgID,rinf] = optargs{:};
% initialize
NumSDOF=length(T);
Sd=zeros(NumSDOF,1);
Sv=zeros(NumSDOF,1);
Sa=zeros(NumSDOF,1);
% Set the eigenfrequencies of the SDOF population
omega=2*pi./T;
% Flip eigenfrequency vector in order for the half-stepping algorithm
% (HalfStep function) to work from large to small eigenperiods
omega=omega(end:-1:1);
% set initial conditions
u0=0;
ut0=0;
% zero-order displacement & velocity overshooting behavior and
% optimal numerical dissipation and dispersion
rinf=1; % mid-point rule a-form algorithm
for j=1:length(T)
omegaj=omega(j);
% Check if dt/T>dtTol. If yes, then reproduce the time history
% with the half step
if dt*omegaj/(2*pi)>dtTol
xgtt=HalfStep(xgtt);
dt=dt/2;
end
[u,ut,utt] = LIDA(dt,xgtt,omegaj,ksi,u0,ut0,AlgID,rinf);
% output
Sd(j)=max(abs(u));
Sv(j)=max(abs(ut));
Sa(j)=max(abs(utt));
end
% Flip output quantities to be compatible with omega
omega=omega(end:-1:1);
Sd=Sd(end:-1:1);
Sv=Sv(end:-1:1);
Sa=Sa(end:-1:1);
% Calculate pseudovelocity and pseudoacceleration
PSv=Sd.*omega;
PSa=Sd.*omega.ˆ2;
end

It can be seen that the initial conditions for all SDOF systems that are analyzed for the generation of the LEReSp are zero for both velocity (${\dot{u}}_{0}$) and displacement ($u_{0}$). Furthermore, the time integration algorithm that is used for the dynamic response history analysis of each SDOF system has zero order overshooting behavior for both displacement and velocity, and since the minimum absolute value of the eigenvalues of the amplification matrix (rinf) is equal to unity, this corresponds to the mid-point rule a-form algorithm.

Three categories of time integration algorithms can be distinguished based on various algorithmic properties: (i) zero-order displacement and velocity overshoot algorithms (U0-V0); (ii) zero-order displacement and first-order velocity overshoot algorithms (U0-V1); and (iii) first-order displacement and zero-order velocity overshoot algorithms (U1-V0). The formulation of the general case of a GSSSS algorithm involves the determination of 12 sets of parameters, which are presented in [39]. Therefore, the user has the freedom to configure the general algorithm by adjusting the time integration constants so that it yields acceptable results for any specific case of application. This capability is also incorporated in OpenSeismoMatlab, since this general time integration framework is an integral part of it. It has been shown that many known time integration algorithms (e.g. the members of the Newmark family) are special cases of this general algorithm framework. A more complete presentation and investigation of the entire time integration algorithm family used in OpenSeismoMatlab can be found in [27].

The function LIDA (Linear Implicit Dynamic Analysis) is utilized for the dynamic analysis of each SDOF system. Initially, the time integration constants are calculated so that the time integration scheme corresponds to a zero-order displacement & velocity overshooting behavior and optimal numerical dissipation and dispersion algorithm. The desired properties of this algorithm are unconditional stability, second-order accuracy, dissipative with optimal dissipation and dispersion, and no overshoot. The minimum absolute value of the eigenvalues of the amplification matrix at the high-frequency limit is imposed to be equal to unity to minimize the effect of the spurious root at the low-frequency limit. The reader is referred to [39] for more details. The default time integration algorithm of OpenSeismoMatlab (mid-point rule a-form algorithm) can be used for small-scale (non-stiff) undamped problems. Of course, in special cases, e.g. for large scale (stiff) problems with initial displacement or initial velocity, the U1-V0 or the U0-V1 algorithms can be used, respectively. In such cases, the various integration constants can be easily adjusted by the user of OpenSeismoMatlab by appropriate modification of the code, so that solutions of superior quality can be obtained. The above, as well as the internal code of the function LIDA are shown in the following:

**Table:** 

function [u,ut,utt] = LIDA(dt,xgtt,omega,varargin)
% set defaults for optional inputs
optargs = {0.05,0,0,1};
% skip any new inputs if they are empty
newVals = cellfun(@(x) ∼isempty(x), varargin);
% overwrite the default values by those specified in varargin
optargs(newVals) = varargin(newVals);
% place optional args in memorable variable names
[ksi,u0,ut0,rinf] = optargs{:};
% Integration constants
% zero-order displacement & velocity overshooting behavior and
% optimal numerical dissipation and dispersion
w1=-15*(1-2*rinf)/(1-4*rinf); % suggested
w2=15*(3-4*rinf)/(1-4*rinf); % suggested
w3=-35*(1-rinf)/(1-4*rinf); % suggested
W1=(1/2+w1/3+w2/4+w3/5)/(1+w1/2+w2/3+w3/4); % definition
W1L1=1/(1+rinf);
W2L2=1/2/(1+rinf);
W3L3=1/2/(1+rinf)ˆ2;
W1L4=1/(1+rinf);
W2L5=1/(1+rinf)ˆ2; % suggested
W1L6=(3-rinf)/2/(1+rinf);
l1=1;
l2=1/2;
l3=1/2/(1+rinf);
l4=1;
l5=1/(1+rinf);

The dynamic response history analysis is performed through the use of the fast MATLAB function *filter*. This function proves to be much faster (up to 100x) than the ordinary time integration routines, and filters the data in any input vector (i.e. the time history of acceleration) with a rational transfer function described by two additional input vectors (denominator and nominator) to create the filtered output data (i.e. the time history of the response). The transfer function of the *filter* function is of the form presented in Eq. (9):

(9) $$Y\left(z\right)=\frac{TF_{n}\left(1\right)+TF_{n}\left(2\right)z^{-1}+TF_{n}\left(3\right)z^{-2}+TF_{n}\left(4\right)z^{-3}}{1+TF_{d}\left(1\right)z^{-1}+TF_{d}\left(2\right)z^{-2}+TF_{d}\left(3\right)z^{-3}}X\left(z\right)$$

where X(z) is the input signal (i.e. the time history of acceleration), Y(z) is the output signal (i.e. the time history of the SDOF dynamic response), TF_n_ is a row vector containing the coefficients of the nominator of the transfer function and TF_d_ is a row vector containing the coefficients of the denominator of the transfer function, as presented in the following.

The calculation of the transfer function denominator and nominator is shown in the source code of the function LIDA.m. The elements of the amplification matrix are calculated first, and then the invariants of the amplification matrix are found as shown in Eqs. (10), (11), (12), and (13):

(10) $$A=\left[\begin{array}{ccc} 1-\frac{\lambda_{3}\Omega^{2}}{D} & \lambda_{1}-\frac{\lambda_{3}\left(2\xi \Omega +\mu_{1}\Omega^{2}\right)}{D} & \lambda_{2}-\lambda_{3}\frac{1+2\mu_{4}\xi \Omega +\mu_{2}\Omega^{2}}{D}\\ -\frac{\lambda_{5}\Omega^{2}}{D} & 1-\frac{\lambda_{5}\left(2\xi \Omega +\mu_{1}\Omega^{2}\right)}{D} & \lambda_{4}-\lambda_{5}\frac{1+2\mu_{4}\xi \Omega +\mu_{2}\Omega^{2}}{D}\\ -\frac{\Omega^{2}}{D} & -\frac{2\xi \Omega +\mu_{1}\Omega^{2}}{D} & 1-\frac{1+2\mu_{4}\xi \Omega +\mu_{2}\Omega^{2}}{D} \end{array}\right]$$

(11) $$I_{A}=tr\left(A\right)=\sum_{i=1}^{3}A_{ii}$$

(12) $$II_{A}=\frac{1}{2}\left[tr{\left(A\right)}^{2}-tr\left(A^{2}\right)\right]$$

(13) $$III_{A}=\det\left(A\right)$$

where Ω=ωΔt is the normalized circular eigenfrequency and $D=\mu_{6}+2\mu_{5}\xi \;\Omega +\mu_{3}\Omega^{2}$, while λ_i_, μ_i_ (i = 1,...,5) and W_1_ are constants of the time integration algorithm. The reader is referred to [27] for the detailed definitions of these integration constants corresponding to the various time integration algorithms. The denominator of the transfer function is given by Eq. (14):

(14) $$TF_{d}=\left[1-I_{A},II_{A},-III_{A}\right]$$

The nominator of the transfer function is calculated as shown in Eq. (15):

(15) $$TF_{n}=\left[B_{1}\text{,}B_{2}\text{,}B_{3}\text{,}B_{4}\right]$$

where B_1_, B_2_, B_3_ and B_4_ are given by Eq. (16):

(16) $$\begin{array}{l} B_{1}=\frac{\Delta t^{2}\lambda_{3}W_{1}}{D}\\ B_{2}=\frac{\Delta t^{2}\left[\lambda_{3}\left(1-W_{1}\right)-\left(A_{22}+A_{33}\right)\lambda_{3}W_{1}+A_{12}\lambda_{5}W_{1}+A_{13}W_{1}\right]}{D}\\ B_{3}=\frac{\Delta t^{2}}{D}\left\{\begin{array}{l} -\left(A_{22}+A_{33}\right)\lambda_{3}\left(1-W_{1}\right)+A_{12}\lambda_{5}\left(1-W_{1}\right)+A_{13}\left(1-W_{1}\right)+\left(A_{22}A_{33}-A_{23}A_{32}\right)\lambda_{3}W_{1}-\\ \left(A_{12}A_{33}-A_{13}A_{32}\right)\lambda_{5}W_{1}+\left(A_{12}A_{23}-A_{13}A_{22}\right)W_{1} \end{array}\right\}\\ B_{4}=\frac{\Delta t^{2}}{D}\left\{\begin{array}{l} \left(A_{22}A_{33}-A_{23}A_{32}\right)\lambda_{3}\left(1-W_{1}\right)-\left(A_{12}A_{33}-{A_{1}}_{3}A_{32}\right)\lambda_{5}\left(1-W_{1}\right)+\\ \left(A_{12}A_{23}-A_{13}A_{22}\right)\left(1-W_{1}\right) \end{array}\right\} \end{array}$$

The denominator vector is constructed by the amplification matrix invariants. The nominator vector is constructed as a function of the elements of the amplification matrix and various integration constants. Finally, the displacement of the SDOF system is found at the first and second time instants by using the initial conditions and the amplification matrix. The time history of the displacement is found using the Matlab function *filter*. Then the velocity is calculated from the system of Eqs. (17) and (18), in which the only unknowns are the time histories of the velocity and acceleration (${\dot{u}}^{t}$ and ${\ddot{u}}^{t}$, respectively):

(17) $$u^{t+\Delta t}={\left[\begin{array}{c} 1-\frac{\lambda_{3}\Omega^{2}}{D}\\ \lambda_{1}-\frac{\lambda_{3}\left(2\xi \Omega +\mu_{1}\Omega^{2}\right)}{D}\\ \lambda_{2}-\lambda_{3}\frac{1+2\mu_{4}\xi \Omega +\mu_{2}\Omega^{2}}{D} \end{array}\right]}^{T}\left[\begin{array}{c} u^{t}\\ \Delta t{\dot{u}}^{t}\\ \Delta t{\ddot{u}}^{t} \end{array}\right]+\frac{\lambda_{3}\Delta t^{2}}{D}\left[\left(1-W_{1}\right)\left(-{\ddot{u}}_{g}^{t}\right)+W_{1}\left(-{\ddot{u}}_{g}^{t+\Delta t}\right)\right]$$

(18) $$m{\ddot{u}}^{t}+c{\dot{u}}^{t}+ku^{t}=-m{\ddot{u}}_{g}^{t}$$

and the acceleration time history, with the time histories of the displacement and velocity known, is derived merely from Eq. (18). In Eqs. (17) and (18) $u^{t}$is the displacement at time t, ${\ddot{u}}_{g}$ is the earthquake ground acceleration, *m, c* and *k* are the mass, damping coefficient and stiffness of the SDOF system respectively. More details about the source code segment in which this computation is performed can be seen in the following.

**Table:** 

% Transfer function denominator
Omega=omega*dt;
D=W1L6+2.*W2L5.*ksi.*Omega+W3L3.*Omega.ˆ2;
A31=-Omega.ˆ2./D;
A32=-1./D.*(2.*ksi.*Omega+W1L1.*Omega.ˆ2);
A33=1-1./D.*(1+2.*W1L4.*ksi.*Omega+W2L2.*Omega.ˆ2);
A11=1+l3.*A31;
A12=l1+l3.*A32;
A13=l2-l3.*(1-A33);
A21=l5.*A31;
A22=1+l5.*A32;
A23=l4-l5.*(1-A33);
% Amplification matrix
A=[A11 A12 A13;A21 A22 A23;A31 A32 A33];
% Amplification matrix invariants
A1=A(1,1)+A(2,2)+A(3,3);
A2=A(1,1)*A(2,2)-A(1,2)*A(2,1)+A(1,1)*A(3,3)-A(1,3)*A(3,1)+A(2,2)*A(3,3)-...
A(2,3)*A(3,2);
A3=A(1,1)*A(2,2)*A(3,3)-A(1,1)*A(2,3)*A(3,2)-A(1,2)*A(2,1)*A(3,3)+A(1,2)*...
A(2,3)*A(3,1)+A(1,3)*A(2,1)*A(3,2)-A(1,3)*A(2,2)*A(3,1);
% Transfer function denominator
a=[1 -A1 A2 -A3];
% Transfer function nominator
B1=1./D.*dtˆ2.*l3.*W1;
B2=1./D.*dtˆ2.*(l3.*(1-W1)-(A22+A33).*l3.*W1+A12.*l5.*W1+A13.*W1);
B3=1./D.*dtˆ2.*(-(A22+A33).*l3.*(1-W1)+A12.*l5.*(1-W1)+A13.*(1-W1)+...
(A22.*A33-A23.*A32).*l3.*W1-(A12.*A33-A13.*A32).*l5.*W1+(A12.*A23-...
A13.*A22).*W1);
B4=1./D.*dtˆ2.*((A22.*A33-A23.*A32).*l3.*(1-W1)-(A12.*A33-A13.*A32).*l5.*(1-...
W1)+(A12.*A23-A13.*A22).*(1-W1));
b=[B1,B2,B3,B4];
% form initial conditions for filter function
% equivalent external force
f=-xgtt;
% stiffness
k=omega.ˆ2;
% damping constants
c=2.*omega.*ksi;
% initial acceleration
utt0=-f(1)-(k*u0+c*ut0);
U_1=A∖[u0;dt*ut0;dtˆ2*utt0];
u_1=U_1(1);
U_2=A∖U_1;
u_2=U_2(1);
ypast=[u0,u_1,u_2];
vinit=zeros(1,3);
vinit(3:-1:1) = filter(-a(4:-1:2),1,ypast);
% main dynamic analysis
u=filter(b,a,f,vinit);
% calculate velocity from the following system of equations:
% 1st: the first scalar equation of the matrix equation (60) in X.Zhou &
% K.K.Tamma (2004)
% 2nd: equation of motion
C_u=omegaˆ2*A(1,3)*dtˆ2-A(1,1);
C_f=-A(1,3)*dtˆ2;
C_ut=A(1,2)*dt-A(1,3)*dtˆ2*2*ksi*omega;
L=1/D*l3*dtˆ2*((1-W1)*[0;f(1:end-1)]+W1*f);
ut=(u+C_u*[u0;u(1:end-1)]+C_f*[0;f(1:end-1)]-L)/C_ut;
% calculate acceleration from equation of motion
utt=-omegaˆ2*u-2*ksi*omega*ut;
end

**5** **Constant Ductility inelastic Response Spectrum**

The Constant Ductility Response Spectrum (CDReSp) is the nonlinear counterpart of the linear elastic response spectrum that is described in the previous section. It is a plot of the peak value of any response quantity as a function of the small strain natural vibration period (T_n_) or frequency (f_n_) of a population of inelastic (bilinear elastoplastic) SDOF systems. Each CDReSp curve is associated with a fixed critical damping ratio ξ and target ductility μ_t_. A number of iterations are performed for each SDOF system (i.e. for each eigenfrequency) of the CDReSp, as the way to determine the yield limit of a SDOF structure based on its dynamic response (ductility) is not straightforward. During the iterations the yield limit is continuously adjusted so that the ductility that is calculated is as close as possible to the target ductility. This fact renders the calculation of the CDReSp more computationally expensive than the calculation of the simple LEReSp. Given its large extent, the related MATLAB code for this function as well as the children (called) functions are not presented here but can be easily found online in the OpenSeismoMatlab package of source files at the File Exchange service of MATLAB Central, or at other repositories. Inside the function CDReSp, a series of dynamic analyses of the linear elastic and the bilinear elastoplastic SDOF system are performed, as can be seen in the following flowchart of the calculation of the CDReSp of an earthquake strong ground motion:

**Table:** 

**Input:**${\ddot{u}}_{g}$,ω,ξ,$\mu_{t}$
InitializeSD,SV,SA,PSV,PSA
Setm,$n_{NR}$,$u_{0}$and${\dot{u}}_{0}$
for each SDOF *i* with eigenfrequency$\omega_{i}$
Find the low strain stiffness$k_{hi}=m\omega_{i}^{2}$
if$\omega_{i}\Delta t/\left(2\pi \right)>0.02$
Reproduce${\ddot{u}}_{g}$with half time step (fromΔttoΔt/2)
SetΔt=Δt/2
end
Perform dynamic analysis of linear elastic SDOF (${\ddot{u}}_{g}$,$k_{hi}$,m,ξ,$u_{0}$,${\dot{u}}_{0}$)
$u_{peak}=\max\left(\left|u\left(t\right)\right|\right)$
$f_{peak}=k_{hi}u_{peak}$
$pos=u_{peak}$
$neg=\frac{u_{peak}}{1.5\mu_{t}}$
$tol_{1}=tol$
for k from 1 to$n_{NR}$
Perform dynamic analysis of bilinear elastoplastic SDOF (${\ddot{u}}_{g}$,$k_{hi}$,m,ξ,$u_{0}$,${\dot{u}}_{0}$,$u_{y,k}$)
$u_{peak}^{NL}=\max\left(\left|u^{NL}\left(t\right)\right|\right)$
$\mu =u_{peak}^{NL}/u_{y,k}$
$res_{k}=\mu_{t}-\mu$
if$\left|res_{k}\right|/\mu_{t}<tol_{1}$
break
else ifk≥2
Adjust$tol_{1}$depending on the number of iterations k
Findpos,neg,$u_{y,k+1}$depending on the sign of$res_{k}$,$res_{k-1}$and$res_{k}-res_{k-1}$
else ifk=1
$u_{y,k+1}=neg$
end
end
Calculate$SD\left(i\right)=\max\left(\left|u^{NL}\left(t\right)\right|\right)=u_{peak}^{NL}$,$SV\left(i\right)=\max\left(\left|{\dot{u}}^{NL}\left(t\right)\right|\right)$and$SA\left(i\right)=\max\left(\left|{\ddot{u}}^{NL}\left(t\right)\right|\right)$
Calculate$PSV\left(i\right)=SD\left(i\right)\omega_{i}$and$PSA\left(i\right)=SD\left(i\right)\omega_{i}^{2}$
end
**Output**:SD,SV,SA,PSV,PSA

For these dynamic analyses, an appropriate external function is called. The flowchart of this function can be found in [27]. It is noted that the constant-ductility inelastic response spectrum is computed through nonlinear dynamic analyses of elastoplastic hysteretic systems, rather than through the simplified approaches that have been proposed in the literature [28, 29, 30]. In the above pseudocode *μ* is the ductility ratio achieved for dynamic nonlinear analysis of a SDOF system, *f*_*peak*_ is the maximum linear elastic force, *n*_*NR*_ is the Newton Raphson number of iterations for convergence to the target ductility, *u*_*peak*_ is the maximum value of the absolute displacement time history of a linear SDOF system, $u_{y,k}$ is the yield limit of a SDOF system at k^th^ iteration, $u^{NL}$ is the displacement time history of the bilinear elastoplastic SDOF system and $u_{peak}^{NL}$ is the maximum value of the absolute displacement time history of a nonlinear SDOF system.

**6** **Fourier Amplitude Spectrum**

The Fourier Amplitude Spectrum (FASp) shows how the amplitude of the strong ground motion varies with frequency. It expresses generally the frequency content of a ground motion and useful information can be extracted from it. The MATLAB code that is used for the calculation of the FASp is shown in the following:

**Table:** 

% FOURIER AMPLITUDE SPECTRUM
[f,U]=FASp(dt,xgtt);
seismic.FAS = U;
seismic.freq = f;

The function FASp.m performs the majority of the calculations needed for the creation of the Fourier Amplitude Spectrum. The highest frequency that is considered for the calculation of the FASp is the Nyquist frequency of the input acceleration (the last is denoted in the code as xgtt). The Fourier spectrum that is calculated is single-sided and based on the MATLAB function fft which applies the Fast Fourier Transform (based on a library called FFTW [40, 41]). The transform length n_FFT_ has been set equal to the minimum power of 2 that gives result larger than the length of the input acceleration. This selection can increase the performance of fft. The Fast Fourier Transform is implemented as shown in Eqs. (19) and (20):

(19) $$U\left(k\right)=\sum_{j=1}^{n_{FFT}}{\ddot{u}}_{g}\left(j\right)W_{n_{FFT}}^{\left(j-1\right)\left(k-1\right)}$$

where

(20) $$W_{n_{FFT}}=e^{\left(-2\pi i\right)/n_{FFT}}$$

is a $n_{FFT}$^th^ root of unity, *U* is the Fourier amplitude, $n_{FFT}$ is the transform length of the fft function and *i* in Eq. (20) is the square root of -1. The MATLAB source code for the function FASp.m is shown in the following:

**Table:** 

function [f,U] = FASp(dt,xgtt)
% Nyquist frequency (highest frequency)
Ny = (1/dt)/2;
% number of points in xgtt
L = length(xgtt);
% Next power of 2 from length of xgtt
NFFT = 2ˆnextpow2(L);
% frequency spacing
df = 1/(NFFT*dt);
% Fourier amplitudes
U = abs(fft(xgtt,NFFT))*dt;
% Single sided Fourier amplitude spectrum
U = U(2:Ny/df+1);
% frequency range
f = linspace(df,Ny,Ny/df)';
end

**7** **Mean Period**

The mean period ($T_{m}$) is computed from the Fourier amplitude spectrum of an acceleration time history by Eq. (21) [42]:

(21) $$T_{m}=\frac{\sum_{i}U_{i}^{2}\left(1/f_{i}\right)}{\sum_{i}U_{i}^{2}}$$

for $0.25Hz\leq f_{i}\leq 20Hz$ with Δf≤0.05Hz. $U_{i}$ is the Fourier amplitude, $f_{i}$ is the frequency corresponding to $U_{i}$ and Δf is the frequency interval. Eq. (21) defines that the mean period is the weighted average of the periods of the spectrum with weighting based on squared Fourier amplitudes. The MATLAB source code that is used for the calculation of $T_{m}$ is presented in the following:

**Table:** 

% MEAN PERIOD
fi = f(f>0.25 & f<20);
Ci = U(f>0.25 & f<20);
Tm = ((Ci(:)’.ˆ2)*(1./fi(:)))/(Ci(:)'*Ci(:));
seismic.Tm = Tm;

## 3 Results & discussion

**Fig. 2:**
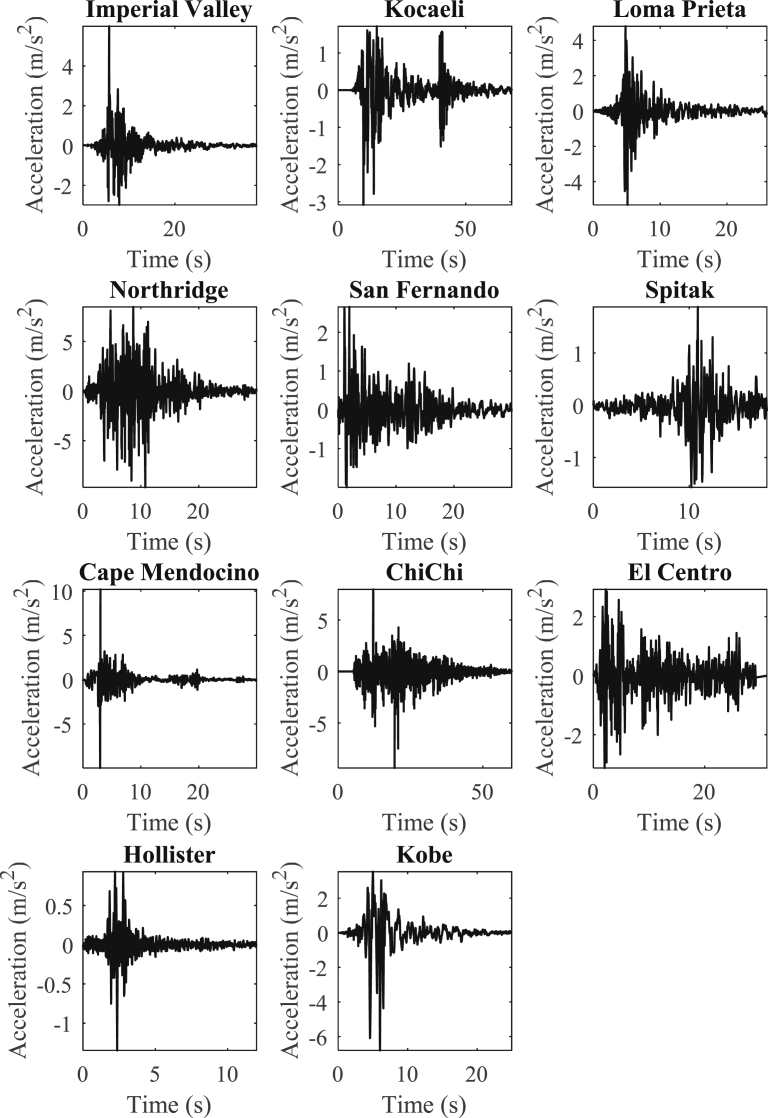
Acceleration time histories of the earthquake records considered.

In order to verify the results of OpenSeismoMatlab, they are compared with the corresponding results of a commercial strong ground motion data processing software, SeismoSignal. A detailed description of this software is given in Section 1. The reason for the selection of this software is that it is easy to use, it has a relatively detailed documentation of high scientific quality and it is accepted as a trustworthy and reliable tool worldwide, since it has been used and tested flawlessly for a number of years by researchers and professionals. However, SeismoSignal uses conventional time integration algorithms, and in certain cases can be susceptible to errors, especially when time integration algorithms with dissipative and overshooting properties superior to those of the Newmark family of algorithms need to be used. OpenSeismoMatlab comes to improve this inadequacy. For the purposes of comparison, a suite of 11 strong ground motion acceleration time histories have been selected, which are presented in Fig. 2. Data about the earthquakes that generated these acceleration time histories are shown in Table 1.

1 **Peak values of displacement, velocity and acceleration time histories**

**Table 1:** Earthquakes the strong motion records of which have been considered in the present study.

Earthquake	Year	Station
Imperial Valley	1979	El Centro Array Sta 8, CA, 95 E Cruickshank Rd
Izmit-Kocaeli	1999	Yarimca Petkim
Loma Prieta	1989	Gilroy Array Sta 3, CA, Sewage Plant
Northridge	1994	090 CDMG Station 24278
San Fernando	1971	Castaic, CA, Old Ridge Route
Spitak	1988	Gukasyan
Cape Mendocino	1992	Cape Mendocino, CA, Petrolia
Chi-Chi	1999	Nantou – Hsinjie School, WNT
El Centro	1940	El Centro Terminal Substation Building
Hollister	1961	USGS Station 1028
Kobe	1995	Takarazuka

In Table 2 the peak ground displacement (PGD), peak ground velocity (PGV) and peak ground acceleration (PGA) are calculated for 11 strong ground motions with various characteristics. Compared to the corresponding results of SeismoSignal (not shown here), it has been observed that the peak values are almost identical with the largest relative difference within 0.5% of the original value.

2 **Arias Intensity values and time histories**

**Table 2:** PGD, PDV and PGA values of the strong motion data considered in this study.

Earthquake	PGD (m)	PGV (m/s)	PGA (m/s^2^)
Imperial Valley	1.233	0.553	5.997
Kocaeli	1.538	0.885	3.085
Loma Prieta	0.106	0.364	5.317
Northridge	0.402	0.787	9.707
San Fernando	1.722	0.337	2.654
Spitak	5.801	0.667	1.879
Cape Mendocino	0.349	0.445	10.194
ChiChi	0.422	0.644	9.373
El Centro	0.212	0.363	3.128
Hollister	0.003	0.042	1.347
Kobe	0.267	0.685	6.803

In Table 3 the Arias intensity is shown for the same 11 strong ground motion records of Table 2. The results of OpenSeismoMatlab coincide with those of SeismoSignal.

**Table 3:** Arias Intensity (AI) of the strong motion data considered in this study.

Earthquake	Arias Intensity (m/s)
Imperial Valley	1.582
Kocaeli	1.669
Loma Prieta	2.075
Northridge	16.634
San Fernando	0.973
Spitak	0.311
Cape Mendocino	2.386
ChiChi	7.569
El Centro	1.802
Hollister	0.044
Kobe	3.067

**Fig. 3:**
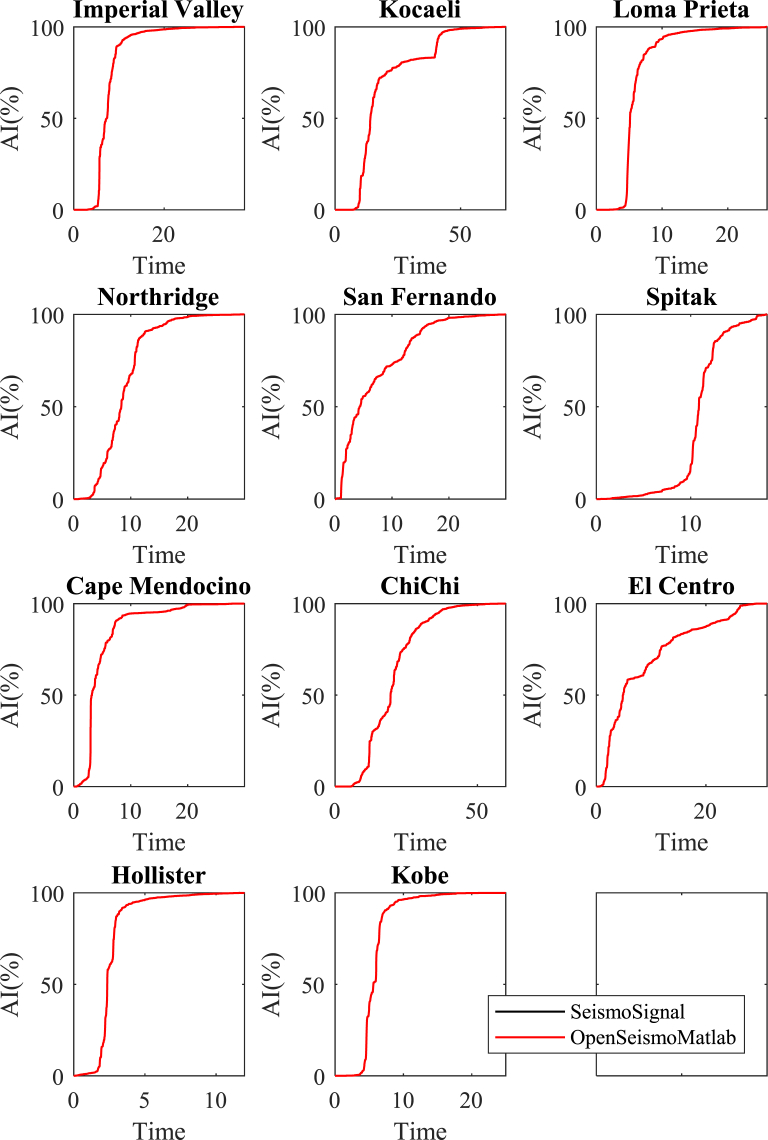
Time histories of the normalized Arias Intensity for the strong motion data considered in this study calculated by OpenSeismoMatlab and SeismoSignal.

In Fig. 3 the time histories of the normalized Arias Intensity (AI) are presented for the 11 strong ground motions considered in this study. The normalization is made with respect to the AI values shown in Table 3. Two time histories are shown in each subplot of this figure. The curves in red color correspond to the results of OpenSeismoMatlab whereas the curves in black color (almost invisible since they are almost fully covered by the red curves) correspond to the results of SeismoSignal. It can be seen that the curves are nearly identical; this shows that the agreement between the two software is very good.

**3** **Significant durations 5-95**

The significant duration of each strong ground motion record are shown in Table 4. The significant duration 5–95 is defined as the time interval between the point at which 5% of the Arias intensity is attained, and the point at which 95% of the Arias intensity is attained. The results of the two programs coincide.

**4** **Elastic response spectra**

**Table 4:** Significant duration of the strong motion data considered in this study.

Earthquake	Significant duration 5–95 (s)
Imperial Valley	6.84
Kocaeli	31.66
Loma Prieta	6.00
Northridge	12.58
San Fernando	15.82
Spitak	8.08
Cape Mendocino	10.04
ChiChi	27.34
El Centro	23.84
Hollister	2.48
Kobe	4.60

**Fig. 4:**
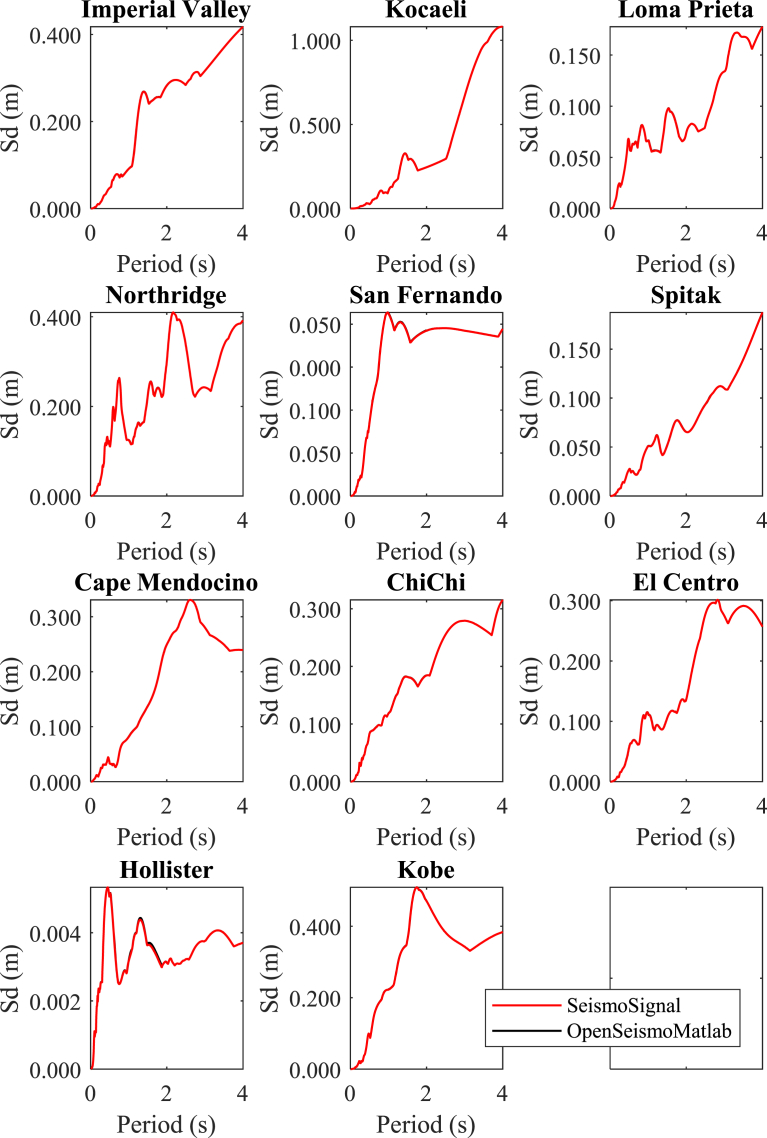
Linear elastic displacement response spectra for the strong motion data considered in this study calculated by OpenSeismoMatlab and SeismoSignal.

**Fig. 5:**
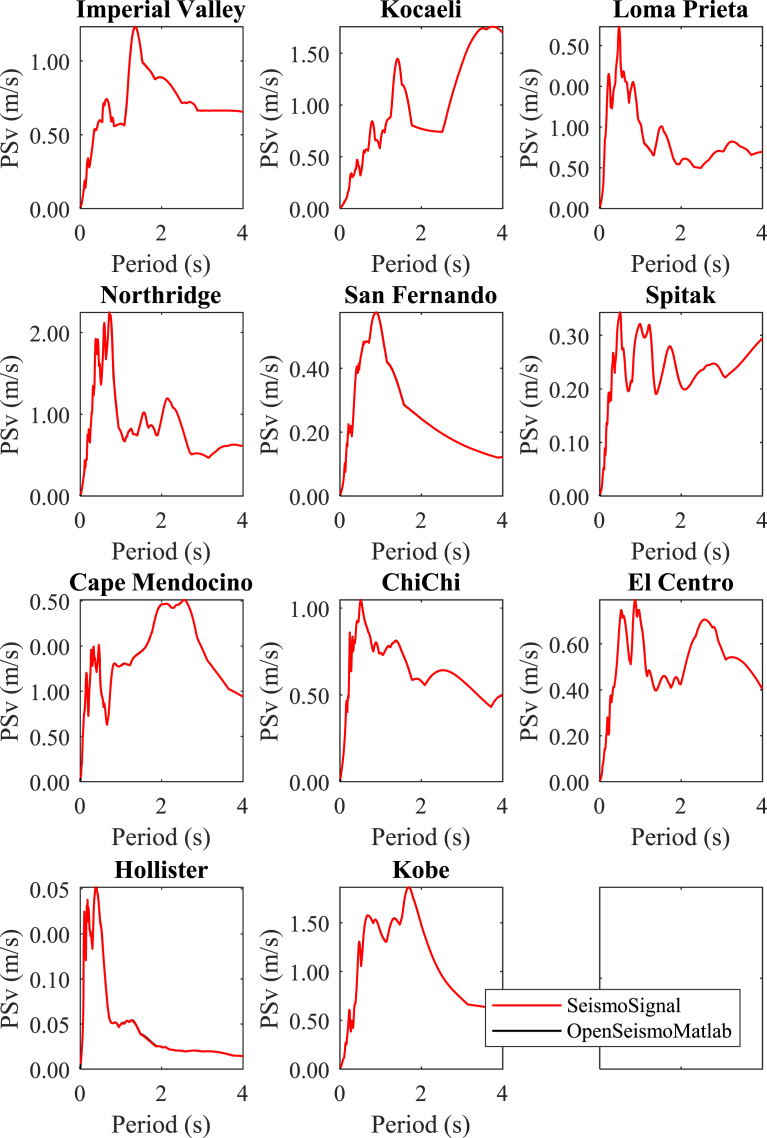
Linear elastic pseudo-velocity response spectra for the strong motion data considered in this study calculated by OpenSeismoMatlab and SeismoSignal.

**Fig. 6:**
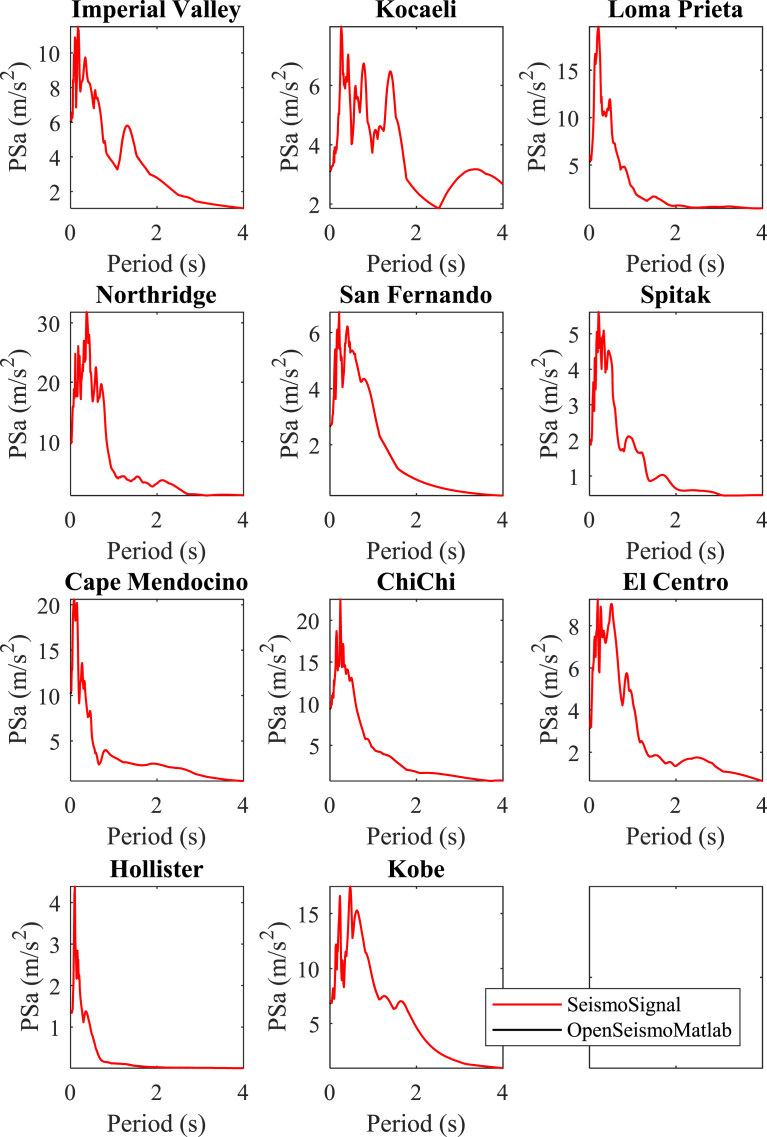
Linear elastic pseudo-acceleration response spectra for the strong motion data considered in this study calculated by OpenSeismoMatlab and SeismoSignal.

In Figs. 4–6 the displacement, pseudo-velocity and pseudo-acceleration response spectra are presented for the various strong ground motions considered in this study. As done also in other figures, the curves with red and the black color correspond to the results of OpenSeismoMatlab and SeismoSignal, respectively. The comparison is excellent between the two programs.

**5** **Constant-ductility inelastic response spectra**

**Fig. 7:**
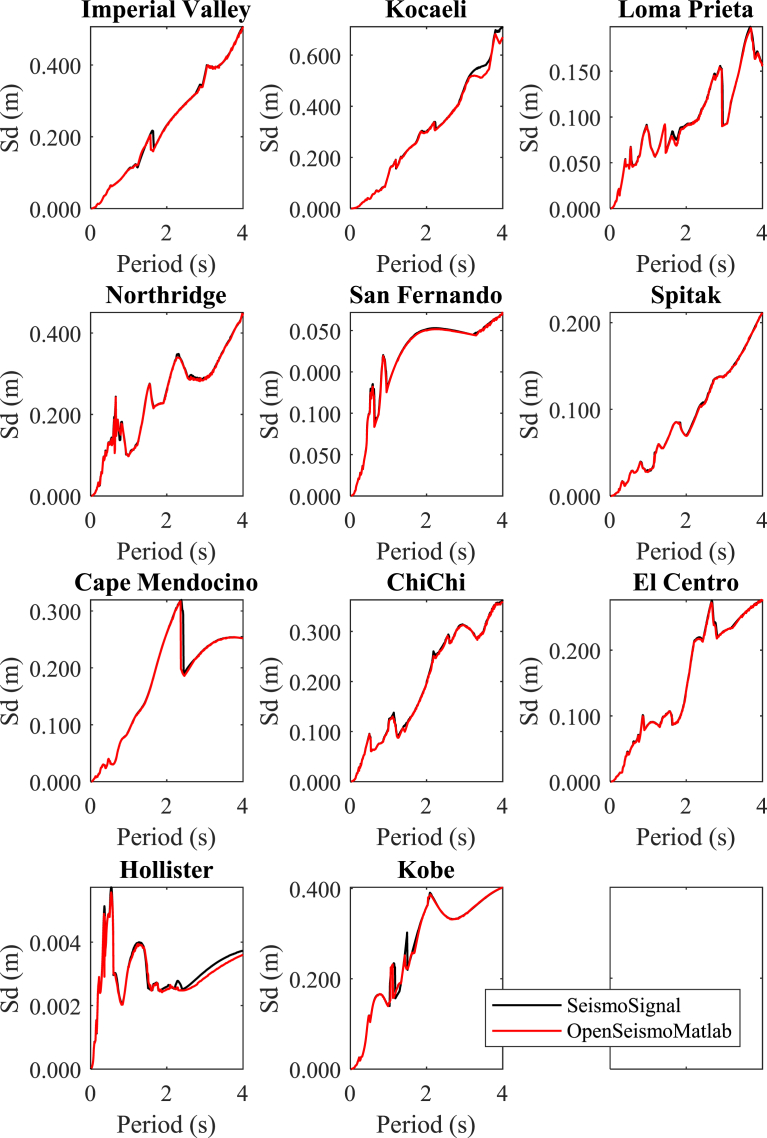
Constant ductility inelastic displacement response spectra for the strong motion data considered in this study calculated by OpenSeismoMatlab and SeismoSignal.

**Fig. 8:**
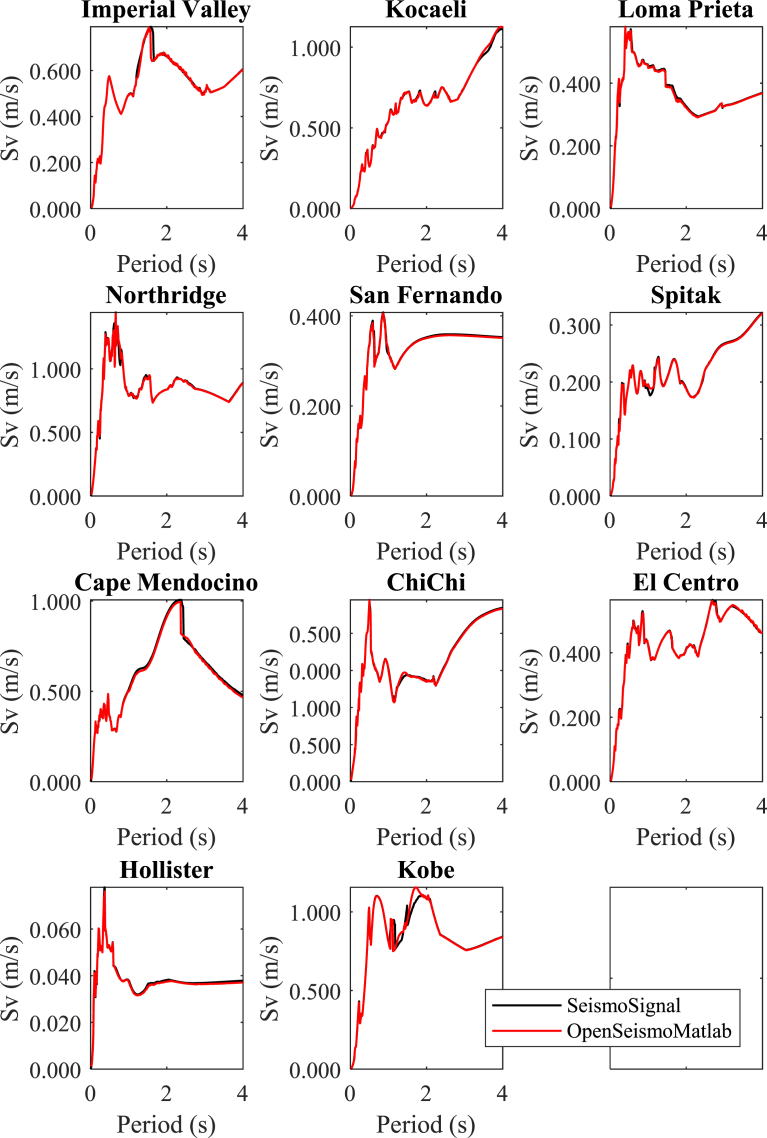
Constant ductility inelastic velocity response spectra for the strong motion data considered in this study calculated by OpenSeismoMatlab and SeismoSignal.

In Figs. 7 and 8 the spectral displacement and spectral velocity respectively are shown for the constant-ductility inelastic response spectra of the 11 strong ground motions considered for target ductility equal to 2. Obviously, the difference between the curves of the results of the two software is larger than that between the linear elastic counterparts. The differences between the results can be attributed to the different methods used by the two software, the superiority of the time integration algorithms used by OpenSeismoMatlab and other factors related to the efficiency of the implementation of the various procedures in the code of the two software. Despite these, generally the corresponding results of the two software are reasonably close to each other also in the nonlinear regime.

**6** **Fourier amplitude spectra**

**Fig. 9:**
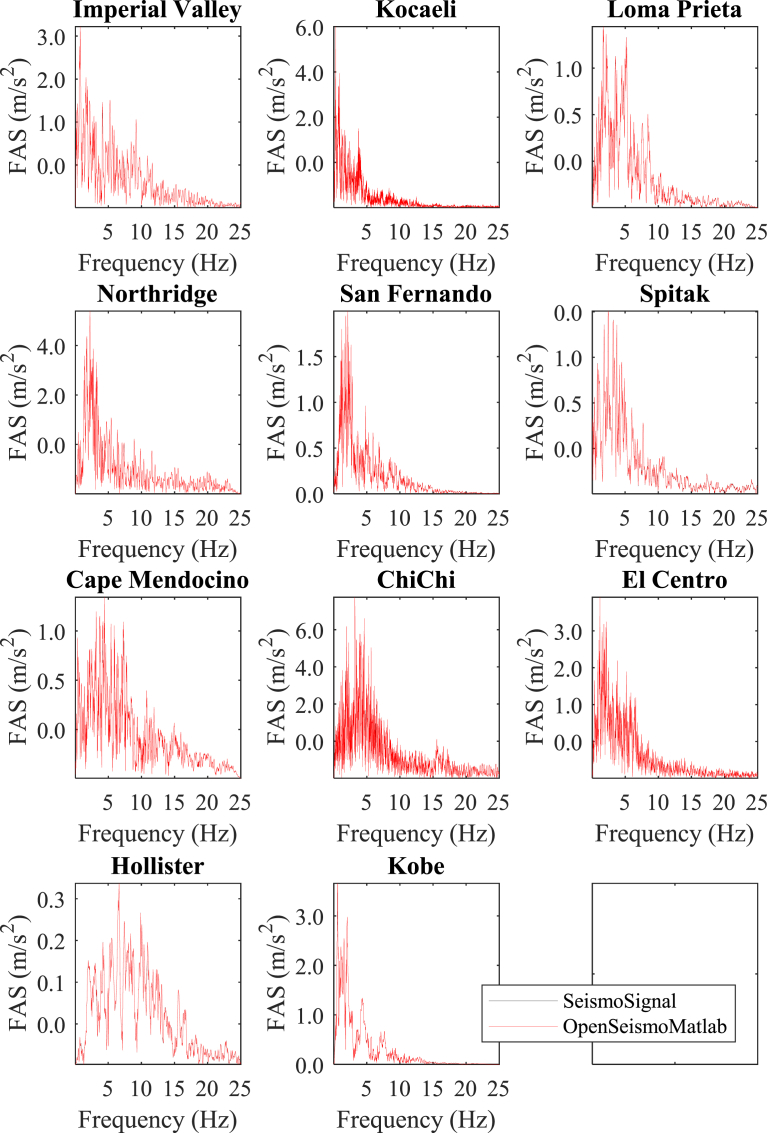
Fourier amplitude spectra for the strong motion data considered in this study calculated by OpenSeismoMatlab and SeismoSignal.

In Fig. 9 the Fourier amplitude spectra (FAS) are shown for the strong ground motions considered. Two curves for each record are shown which correspond to the two software being compared. It seems that the various Fourier spectra are nearly identical.

**7** **Effect of the time step on the accuracy of the output**

**Fig. 10:**
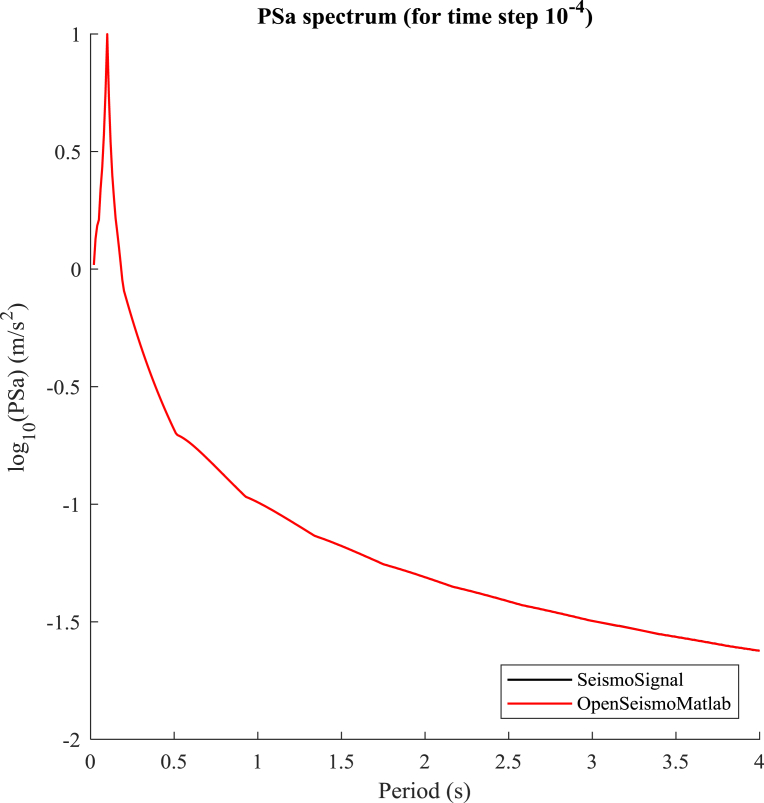
Comparison of the pseudoacceleration response spectrum PSa for a very small step size (Δt = 10^−4^ s) between OpenSeismoMatlab and SeismoSignal.

In this section, the effect of the time step size on the accuracy of the solutions provided by OpenSeismoMatlab is investigated. The pseudoacceleration (PSa) response spectrum of the acceleration time history corresponding to the function ${\ddot{u}}_{g}=\sin\left(20\pi t\right)$with critical damping ratio ξ equal to 5% is considered. The excitation is a harmonic (sinusoidal) motion with circular frequency equal to 20π (i.e. frequency 10 Hz and period 0.1 s) and total duration 2 s, whereas it is digitized in sufficiently small time steps (Δt = 0.0001 s). The PSa spectrum is calculated for OpenSeismoMatlab and SeismoSignal separately and initially a comparison is made between the two solutions. This comparison is shown in Fig. 10, in which the decimal logarithm of the PSa spectrum is plotted versus the range of eigenperiods considered. It is obvious that the two curves nearly coincide with each other and from this it can be concluded that practically they both coincide with the real solution, since the time step is relatively small enabling thus high accuracy computations. The differences between the two solutions are very small, found only at the 5^th^ decimal digit. We define as PSa_0_ this reference solution, i.e PSa_0_ is considered the correct solution for each program.

**Fig. 11:**
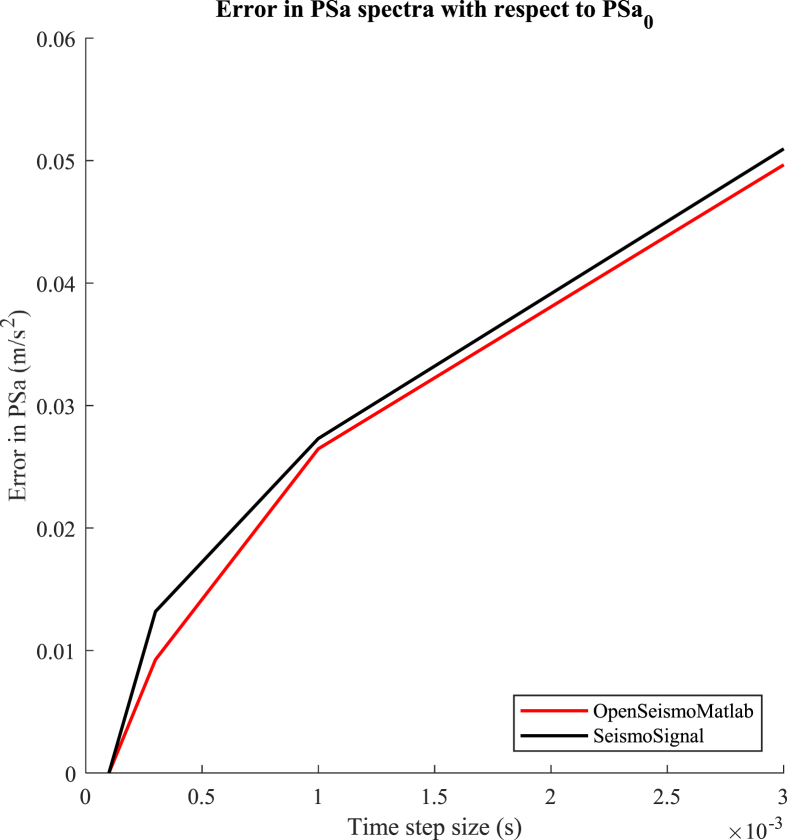
Comparison of the error of the pseudoacceleration spectrum (PSa) with respect to PSa_0_, between OpenSeismoMatlab and SeismoSignal.

As the time step size increases, a certain degree of error is introduced in the PSa spectrum. A measure of this error can be the root-mean-square deviation between the PSa spectrum for an arbitrary value of Δt and the accurate PSa_0_ presented in Fig. 10, which is estimated by Eq. (22) as follows:

(22) $$RMSD=\sqrt{\frac{\sum_{i=1}^{n}{\left(PSa_{\Delta t}^{i}-PSa_{0}^{i}\right)}^{2}}{n}}$$

where PSa_Δt_ is the PSa spectrum obtained for time step equal to Δt and n is the number of eigenperiods contained in the PSa spectrum (n = 400 in this investigation). The different values of Δt that are considered are 3×10^−4^ s, 1×10^−3^ s and 3×10^−3^ s. For each value of these time steps, two PSa_Δt_ spectra are calculated, one by OpenSeismoMatlab and one by SeismoSignal. Then, Eq. (22) is applied for the two programs separately, where for each one the corresponding PSa_0_ is considered; two separate RMSD curves are extracted and plotted in Fig. 11 for comparison. It is obvious that the solutions provided by OpenSeismoMatlab have less error than the corresponding solutions provided by SeismoSignal, for the various time step sizes. As a result, it is shown that the quality of the results of OpenSeismoMatlab is superior to that of SeismoSignal, at least under certain circumstances. This is attributed to the fact that advanced time integration algorithms are used by the former.

## 4 Conclusions

A new open-source software for strong ground motion data processing called OpenSeismoMatlab is presented, which uses advanced time integration algorithms, contains open and free source code written in MATLAB, and uses an accurate formulation and implementation of the elastoplastic bilinear kinematic hardening constitutive model. Parts of the code have been presented and explained in detail in this paper, so that the reader can easily understand the structure and implementation of the software and make various case-dependent adjustments in order to obtain results of the highest quality. The various types of spectra of 11 earthquake strong ground motions have been extracted with OpenSeismoMatlab and it has been shown that they are nearly identical to corresponding results of SeismoSignal, a reliable commercial proprietary software. In some cases, the quality of the results of the new software is superior to that of SeismoSignal due to the fact that it uses advanced time integration algorithms that allow for controlled dissipation, dispersion and overshooting properties. A numerical investigation was made which showed that OpenSeismoMatlab provides more accurate results than SeismoSignal when the same integration step size is used for both. OpenSeismoMatlab is a unique software that combines innovative numerical algorithms, high quality and robustness and is provided as an open-source tool to the research and professional engineering communities for the seismic design of structures as well as the processing of strong ground motions. The new software can be used for free by students and/or programmers for the seismic design of structures as well as general processing of strong ground motions. Thanks to its open source nature, it can be of high educational value for related university courses and can be easily extended or modified in order to be incorporated in higher level software.

## Declarations

### Author contribution statement

George Papazafeiropoulos: Conceived and designed the experiments; Performed the experiments; Wrote the paper.

Vagelis Plevris: Conceived and designed the experiments; Analyzed and interpreted the data; Contributed reagents, materials, analysis tools or data.

### Funding statement

This research did not receive any specific grant from funding agencies in the public, commercial, or not-for-profit sectors.

### Competing interest statement

The authors declare no conflict of interest.

### Additional information

Data associated with this study has been deposited at https://www.mathworks.com/matlabcentral/fileexchange/67069-openseismomatlab.
